# Real-world treatment patterns and visual outcomes of faricimab in patients with neovascular age-related macular degeneration in the UK at 12 months: the FARWIDE-nAMD study

**DOI:** 10.1038/s41433-025-04213-2

**Published:** 2026-03-10

**Authors:** James Talks, Gabriella de Salvo, Praveen J. Patel, Samantha R. de Silva, Richard P. Gale, Martin McKibbin, Deepali Varma, Ian Pearce, Tunde Peto, Rhianon Reynolds, Clare Bailey, Louise Downey, Christine A. Kiire, Sobha Sivaprasad, Amanda K. Downey, Natalee James, Gloria C. Chi, Melanie Dodds, Parul Dayal

**Affiliations:** 1 Newcastle Hospitals NHS Foundation Trust, Newcastle upon Tyne, UK; 2 University Hospital Southampton NHS Foundation Trust, Southampton, UK; 3 University of Southampton, Southampton, UK; 4 NIHR Moorfields Biomedical Research Centre, Moorfields Eye Hospital, London, UK; 5 Oxford University Hospitals NHS Foundation Trust, Oxford, UK; 6 Nuffield Department of Clinical Neurosciences, University of Oxford, Oxford, UK; 7 University of York, York, UK; 8 York and Scarborough Teaching Hospitals NHS Foundation Trust, York, UK; 9 Leeds Teaching Hospitals NHS Trust, Leeds, UK; 10 South Tyneside and Sunderland NHS Foundation Trust, Sunderland, UK; 11 Liverpool University Hospitals NHS Foundation Trust, Liverpool, UK; 12 Queen’s University Belfast, Belfast, UK; 13 Belfast Health and Social Care Trust, Belfast, UK; 14 Aneurin Bevan University Health Board, Newport, UK; 15 Cardiff University School of Vision Science, Cardiff, UK; 16 University Hospitals Bristol NHS Foundation Trust, Bristol, UK; 17 Hull University Teaching Hospitals NHS Trust, Hull, UK; 18 F. Hoffmann-La Roche Ltd, Mississauga, ON Canada; 19 Roche Products Ltd, Welwyn Garden City, UK; 20 Genentech, Inc., South San Francisco, CA USA; 21 Medisoft Limited, Leeds, UK

**Keywords:** Macular degeneration, Retinal diseases

## Abstract

**Background:**

The Faricimab Real-World Evidence (FARWIDE) studies are evaluating real-world outcomes of eyes with neovascular age-related macular degeneration (nAMD) or diabetic macular oedema (DMO) treated with faricimab in the UK. Here, we present results from FARWIDE–nAMD for eyes with 12 months of follow-up after faricimab initiation.

**Methods:**

nAMD patient-eyes that received ≥1 faricimab injection after May 2022 at one of 35 participating UK National Health Service retinal clinics with ≥12 months of follow-up after faricimab initiation as of July 2024 were included. Treatment-naïve (TN) eyes had no prior anti-VEGF treatment. Previously treated (PT) eyes switched from an anti-VEGF to faricimab. Baseline characteristics, VA, and injection frequency were assessed. Intraocular inflammation (IOI) and presumed infectious endophthalmitis (PIE) rates were pooled for nAMD and DMO eyes with any follow-up duration on faricimab. Analyses are descriptive.

**Results:**

5854 nAMD patients (6991 eyes; 26.5% TN, 73.5% PT) were included. 83.3% of PT eyes switched from aflibercept 2.0 mg. TN eyes received a mean (SD) of 4.7 (0.7) faricimab injections in months 1–6 and 2.2 (1.1) injections in months 7–12. PT eyes received 4.5 (1.0) injections in months 1–6 and 3.0 (1.2) in months 7–12. In TN eyes, mean (SD) VA increased from 56.4 (16.3) Early Treatment Diabetic Retinopathy Study letters at baseline to 60.1 (19.4) at 12 months (mean [SD] change 3.6 [14.7] letters). PT eyes had stable VA. IOI and PIE rates were consistent with faricimab phase 3 trials.

**Conclusions:**

These 1-year data support real-world faricimab effectiveness, durability, and safety in nAMD.

## Introduction

Neovascular age-related macular degeneration (nAMD), a major cause of vision loss among adults in developed countries, affects over 200 million people worldwide [1]. nAMD treatment may involve frequent intravitreal injections of anti-vascular endothelial growth factor (VEGF) therapies, including ranibizumab and aflibercept 2.0 mg [2, 3]. Although treatment patterns and clinical outcomes, including long-term vision and retinal stability, are consistent among homogenous groups of carefully selected patients in clinical trials, real-world outcomes are more heterogeneous. This is due to diverse patient demographics, socioeconomic status, disease characteristics, prior treatment history, treatment adherence, patient and provider preferences, local treatment decisions and patient financial constraints [4–8]. Many of these factors can negatively impact patients’ management of the burden of frequent treatment, resulting in suboptimal outcomes. More durable therapies are required to minimise treatment burden. Furthermore, effectiveness and safety of new therapies should be evaluated in real-world practice as outcomes may differ from clinical trials [9].

Faricimab is a humanised, bispecific monoclonal antibody approved for treatment of nAMD in the United States (US), Europe, and the United Kingdom (UK) in 2022 [10–12]. Whereas anti-VEGF therapies reduce pathological neovascularisation associated with nAMD, faricimab addresses the multifactorial nature of the disease by concomitantly stabilising retinal vasculature and reducing inflammation through additional inhibition of angiopoietin-2 (Ang-2) [13]. Faricimab efficacy and safety in treatment-naïve eyes with nAMD were demonstrated in the pivotal TENAYA/LUCERNE phase 3 clinical trials, which randomised 671 and 658 eyes, respectively, to either intravitreal faricimab 6.0 mg with extended dosing up to every 16 weeks or aflibercept 2.0 mg administered every 8 weeks (Q8W) following an initial loading period [14]. Both trials showed non-inferior vision and anatomical improvements for faricimab extended dosing versus aflibercept Q8W during the first year that were maintained during the second year of treatment [14, 15]. Serious ocular adverse event (AE) rates (including intraocular inflammation) were low for both treatments [14, 15]. This suggests that faricimab might be used to extend intravitreal treatment intervals for patients with nAMD, reducing treatment burden.

Although results from the pivotal faricimab trials are supported by observations from increasing numbers of real-world studies from several countries, a majority of these studies are small (<100 patient-eyes), single-centre studies with limited generalisability, reporting early outcomes of faricimab treatment (<6 months) [16–24]. Longer-term studies that include larger and more generalisable populations that evaluate the effectiveness and durability of faricimab for nAMD treatment are required.

Faricimab Real-World Evidence (FARWIDE) is an ongoing multi-centre, retrospective, observational study evaluating real-world treatment patterns and vision outcomes in eyes with nAMD (FARWIDE-nAMD) or diabetic macular oedema (DMO; FARWIDE-DMO) treated with faricimab in the UK. This paper describes results in patient-eyes with nAMD that have at least 12 months follow-up after faricimab initiation.

## Methods

### Objectives

The primary objectives of FARWIDE-nAMD are to evaluate baseline characteristics, treatment frequency, visual outcomes, and safety of faricimab over time among patients with nAMD.

### Study design and data source

FARWIDE-nAMD is an ongoing, retrospective, multi-centre observational study. Patient data were extracted from the Medisoft Ophthalmology (first-generation system) or MediSIGHT (flagship system) electronic medical record systems (Medisoft Limited, Leeds, UK; additional details in Supplementary Methods) used at 35 National Health Service (NHS) trusts with medical retina clinics across the UK (listed in Supplementary Table 1), allowing for data aggregation and standardisation. Data were extracted and analysed by Medisoft Limited. Data were anonymised by removing patient, study site, and clinician identifier, and by perturbing all dates on the patient’s record by a randomly generated number of days (–180 to +180, excluding zero). Data reported were collected between June 2022 and July 2024.

### Patient population

Eyes diagnosed with nAMD that received at least one faricimab injection after May 2022 were included. We required patients to be at least 50 years old at their first faricimab injection. Eyes with concomitant DMO or retinal vein occlusion, or prior or concomitant participation in any clinical trials with faricimab or other intravitreal injections were excluded. Eyes with no recorded baseline visual acuity (VA) or inconclusive treatment history (could not be classified as anti-VEGF treatment-treatment naïve or previously treated at baseline) were excluded. Both patient eyes were included if they met study selection criteria. Results from eyes with ≥12 months of follow-up following faricimab initiation (the “12-month cohort”) are described. Safety is reported for the pooled cohort of nAMD and DMO eyes in FARWIDE with any follow-up duration since faricimab initiation.

### Study variables

#### Baseline patient characteristics

The following demographic characteristics were captured: Age in years at baseline, sex (male, female or not stated), race (White British, White Irish, Asian or Asian British, Black or Black British, any other White background, any other ethnic group, any other mixed background, or not stated; self-reported by patient), Index of Multiple Deprivation (IMD); a small area measure of relative deprivation across the constituent nations of the UK, detailed in Supplement and categorised as deciles ranked from most [1] to least [10] deprived [25, 26].

#### Baseline ocular characteristics

The following ocular characteristics were captured: Laterality of nAMD and faricimab treatment, percentage of anti-VEGF naïve and previously treated patient-eyes (treatment-naïve eyes had no evidence of anti-VEGF injection at any time before baseline; previously treated eyes receive ≥1 prior anti-VEGF treatment at any time pre-baseline at the participating site), and baseline VA (defined as VA captured on or within 28 days before baseline), number of anti-VEGF injections received any time pre-baseline, and number of distinct anti-VEGF agents used (categorised as 1, 2, 3, or >3).

#### Faricimab treatment characteristics

Faricimab injection frequency (number of injections in the first and second 6 months, and overall, 12 months since faricimab initiation) and faricimab loading doses (the number of consecutive faricimab injections that had a treatment interval of 28 ± 14 days, up to the fourth injection) were analysed. Switch from faricimab or initiation of concomitant treatment was defined as the eye receiving at least two doses of an anti-VEGF agent (other than faricimab) or one steroid implant after the date of first faricimab injection. Additional details are provided in the Supplementary Methods.

#### Visual acuity outcomes

VA was measured in clinics using Early Treatment Diabetic Retinopathy Study (ETDRS) letter score, Snellen score (metres), or Logarithm of the Minimum Angle of Resolution (logMAR). Snellen and logMAR values were converted to approximate ETDRS letters for the purposes of this analysis according to Gregori et al. [27]. For VA at 12 months, any measurement recorded within 56 days either side of the 12-month (365 days) time point was selected. If multiple measurements were available, the value closest to baseline or 12-month follow-up date, respectively, was selected. VA was considered a continuous variable, and grouped into the following categories: ≤34, 35-55, 56-69, ≥70 letters. VA change from baseline to 12 months was evaluated and expressed as mean (95% confidence interval [CI]). Attainment and maintenance of VA ≥70 letters (driving level visual acuity) at 12 months were assessed amongst eyes with VA <70 and ≥70 ETDRS letters at baseline, respectively. Gain or avoidance of loss of 10 ETDRS letters was assessed in eyes with VA of ≤90 and ≥10 ETDRS letters at baseline, respectively. Gain or avoidance of loss of 15 ETDRS letters was assessed in eyes with VA of ≤85 and ≥15 ETDRS letters at baseline, respectively.

#### Adverse events

Adverse event analysis was limited to intraocular inflammation and presumed infectious endophthalmitis. Included adverse events were within 180 days after a faricimab injection and before a subsequent faricimab or anti-VEGF injection. If adverse events of the same type occurred within 30 days of each other, only the first was included. One event per injection was counted. Intraocular inflammation and presumed infectious endophthalmitis events were identified using the post-operative complications, diagnoses, or clinical examination findings on the patient record. Presumed infectious endophthalmitis events were additionally identified using surgical records of vitreous biopsy, anterior chamber tap or intravitreal treatment with ceftazidime or vancomycin in the electronic medical record [28]. We report the rate of events per 100 injections (% of events per injection) in the overall FARWIDE population, including nAMD and DMO. The exact χ² method was used to calculate 95% CI for the rates. Additional details, including clinical terms used to define intraocular inflammation and presumed infectious endophthalmitis events, are included in the Supplement.

### Statistical analysis

Analyses are descriptive. Means (standard deviation [SD]) were reported for continuous variables. Frequency and percentages were reported for categorical variables. Additional details are included in the Supplementary Methods.

### Ethical approvals

The study was conducted in accordance with the Declaration of Helsinki and the UK’s Data Protection Act. Use of anonymised patient data was approved by the Caldicott Guardian and the lead clinician for the Medical Retina Service at each site. Informed patient consent was not required.

## Results

### Study population and baseline characteristics

**Fig. 1:**
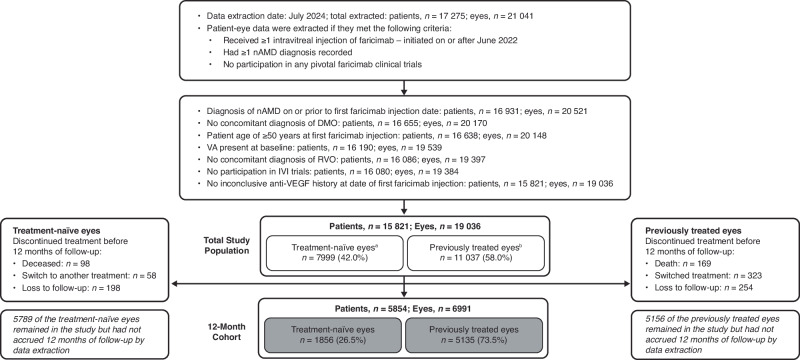
Patient attrition. ^a^ Treatment-naïve eyes are those with no evidence of anti-VEGF injections at any time before initiating faricimab. ^b^ Previously treated eyes are defined as those that received one or more prior anti-VEGF treatment any time before faricimab initiation at the participating site. ^c^ Deceased markers may not be a true reflection of the patient’s vitality status dependent on the Patient Administration System used by the site and its interface with the Medisoft electronic medical records. DMO diabetic macular oedema, IVI intravitreal injection, nAMD neovascular age-related macular degeneration, RVO retinal vein occlusion, VEGF vascular endothelial growth factor.

FARWIDE-nAMD included 15,821 patients (19,036 patient-eyes). Of these, 5854 patients (6991 eyes) completed 12 months of follow-up on faricimab at the time of data extraction (July 2024) and were included in the 12-month cohort. 1856 eyes (26.5%) in the 12-month cohort were treatment naïve and 5135 (73.5%) were previously treated (Fig. 1). 12,045 eyes (6143 treatment-naïve eyes and 5902 previously treated) did not reach 12 months of follow-up at the time of data extraction. Of these, a vast majority of the eyes (10,945 total, 5789 naive, 5156 previously-treated eyes) did not have the opportunity for 12 months to elapse since their index date. The remaining eyes (1100 total, 354 naive, 746 previously-treated) were lost to follow-up, deceased or switched to another treatment before completing 12 months of follow-up on faricimab (Fig. 1).

### 12-month cohort

45.2% had bilateral nAMD diagnosis and 30.0% received bilateral faricimab treatment. Mean (SD) cohort age at baseline was 80.0 (7.8) years for treatment-naïve eyes and 79.8 (7.4) years for previously treated eyes (Table 1). The cohort was mostly female (61.3% treatment-naïve, 57.1% previously treated) and White (66.2% treatment-naïve, 64.3% previously treated). 17.0% of treatment-naïve eyes and 11.1% of previously treated eyes were from patients in the most deprived IMD deciles (1–2) (Table 1).

**Table 1:** Baseline characteristics of the 12-month cohort.

Baseline characteristics	Treatment-naïve eyes (*n* = 1856)	Previously treated eyes (*n* = 5135)
**Age, mean (SD)**	80.0 (7.8)	79.8 (7.4)
**Sex,** ***n*** **(%)**		
Female	1138 (61.3)	2934 (57.1)
Male	662 (35.7)	2118 (41.3)
Not stated	56 (3.0)	83 (1.6)
**Race,** ***n*** **(%)**		
White/White British/White Irish	1230 (66.3)	3301 (64.3)
Asian/Asian British	25 (1.3)	48 (0.9)
Black/Black British	4 (0.2)	7 (0.1)
Other^a^	63 (3.4)	155 (3.0)
Not stated	534 (28.8)	1624 (31.6)
**Index of Multiple Deprivation Deciles,**^**25,26,b**^ ***n*** **(%)**		
1–2 (most deprived)	316 (16.9)	570 (11.1)
3–4	358 (19.2)	730 (14.2)
5–6	390 (21.0)	1052 (20.5)
7–8	398 (21.4)	1233 (24.0)
9–10 (least deprived)	369 (19.9)	1355 (26.4)
Unknown	25 (1.4)	195 (3.8)
**Laterality of disease,** ***n*** **(%)**	316 (16.9)	
Unilateral		2143 (41.7)
Bilateral		2992 (58.3)
**Laterality of faricimab treatment for nAMD,** ***n*** **(%)**		
Unilateral	1256 (67.7)	2841 (55.3)
Bilateral	600 (32.3)	2294 (44.7)
**Baseline VA, ETDRS letters**		
Mean (SD), ETDRS letters	56.4 (16.3)^c^	64.4 (15.1)^d^
Median (Q1, Q3)	60 (46, 70)^c^	68 (55, 76)^d^
**Eyes by baseline VA category (ETDRS letter),** ***n*** **(%)**		
≤34	96 (8.1)^c^	192 (4.2)^d^
35 to 55	432 (36.6)^c^	972 (21.2)^d^
56 to 69	336 (28.5)^c^	1210 (26.4)^d^
≥70	317 (26.8)^c^	2203 (48.1)^d^
**Ocular conditions**		
Glaucoma	112 (6.0)	357 (7.0)
Cataract	498 (26.8)	1867 (36.4)
Amblyopia	8 (0.4)	25 (0.5)
Diabetic retinopathy	25 (1.4)	79 (1.5)
**Lens status**		
Phakic	1173 (63.2)	2952 (57.5)
Pseudophakic	558 (30.1)	2095 (40.8)
Aphakic	2 (0.1)	7 (0.1)
Not stated	123 (6.6)	81 (1.6)
**Prior anti-VEGF treatment characteristics at baseline**		
**Duration of treatment, years**^**e**^		
Mean (SD)	-	2.7 (2.4)
Median (Q1, Q3)		2 (1, 4)
**Total number of injections**		
Mean (SD)	-	22.4 (17.0)
Median (Q1, Q3)		18 (10, 31)
**Number of distinct agents at any time,*n*(%)**		
1		3709 (72.3)
2	-	1250 (24.3)
3		172 (3.4)
>3		4 (0.1)
**Prior treatment,*n*(%)**		
Aflibercept 2.0 mg		4279 (83.3)
Ranibizumab		465 (9.1)
Brolucizumab	-	176 (3.4)
Bevacizumab		69 (1.3)
Ranibizumab biosimilars^f^		146 (2.8)

Baseline is defined as the date of the first faricimab injection.

*ETDRS* Early Treatment Diabetic Retinopathy Study, *LSOA* Lower Layer Super Output Area, *Q* quartile, *SD* standard deviation, *VA* visual acuity, *VEGF* vascular endothelial growth factor.

^a^ Includes any other ethnic group and any other mixed background.

^b^ The deciles are calculated per nation by ranking the 32,844 LSOAs in England and 6976 Data Zones in Scotland from most deprived to least deprived and dividing them into 10 equal groups, respectively. Areas in decile 1 fall within the most deprived 10% and areas in decile 10 fall within the least deprived 10% of LSOAs/Data Zones per nation.

^c^ *n* = 1181 for eyes with VA at baseline and 12 months.

^d^ *n* = 4577 for eyes with VA at baseline and 12 months.

^e^ Number of years between the first anti-VEGF injection and the first faricimab injection. *nAMD* neovascular age-related macular degeneration.

^f^ Ranibizumab biosimilars were Ongavia^®^, and Byooviz™.

At the patient level, faricimab treatment was bilateral in 32.3% of treatment-naïve and 44.7% of previously treated eyes. Cataract was most frequently reported amongst assessed ocular comorbidities in treatment-naïve (26.8%) and previously treated (36.4%) eyes. Pseudophakia was found in 30.1% of treatment-naïve and 40.8% of previously treated eyes.

### Prior treatment history

Amongst previously treated eyes, the mean (SD) duration of anti-VEGF treatment before baseline was 2.7 (2.4) years, and the mean (SD) number of prior injections received overall was 22.4 (17.0). Most eyes received one distinct anti-VEGF agent before faricimab initiation (*n* = 3709, 72.2%), with aflibercept 2.0 mg being the most frequent (83.3%) (Table 1).

### Faricimab treatment: injection frequency

**Fig. 2:**
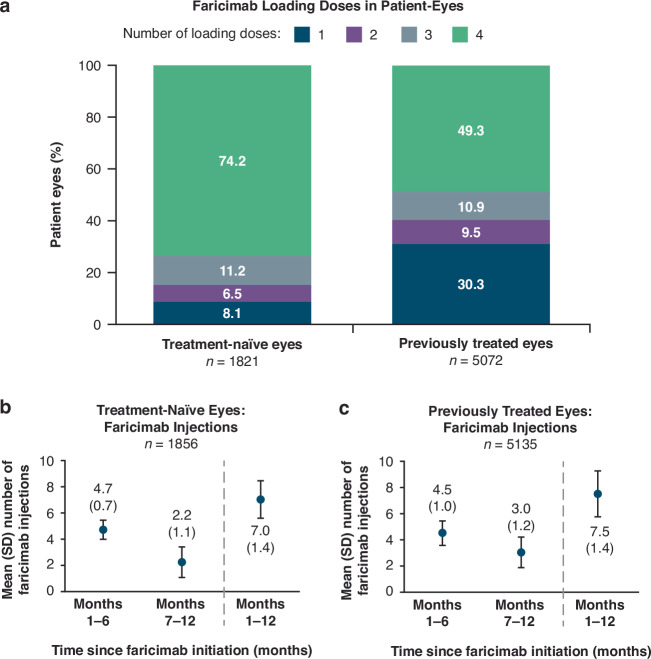
Faricimab loading doses and injection frequency in the 12-month cohort. **a** Loading doses in faricimab treated patient-eyes. Total count is among eyes with four or more faricimab injections. **b** Mean number of faricimab injections in treatment-naïve eyes in months 1–6 and months 7–12 of follow-up. **c** Mean number of faricimab injections in previously treated eyes in months 1–6 and months 7–12 of follow-up. SD standard deviation.

Amongst eyes receiving ≥4 faricimab injections, 74.2% of treatment-naïve eyes and 49.3% of previously treated eyes received four loading doses (Fig. 2a). The mean (SD) number of faricimab injections in treatment-naïve eyes was 4.7 (0.7) in months 1–6 and 2.2 (1.1) in months 7–12 (Fig. 2b), with a mean of 7.0 (1.4) injections throughout the full 12 months. In previously treated eyes, the mean (SD) number of faricimab injections was 4.5 (1.0) in months 1–6 and 3.0 (1.2) in months 7–12 (Fig. 2c) with a mean of 7.5 (1.8) injections during the full 12 months. 0.5% of treatment-naïve eyes and 0.7% of previously treated eyes switched from faricimab to an anti-VEGF treatment.

### Visual acuity outcomes

Amongst eyes with VA measurements at 12 months, mean baseline VA was 56.4 ETDRS letters in treatment-naïve eyes (*n* = 1181) with 26.8% eyes having a baseline VA of ≥70 ETDRS letters. For previously treated eyes (*n* = 4577), mean baseline VA was 64.4 ETDRS letters; 48.1% of eyes had a baseline VA of ≥70 ETDRS letters (Table 1).

**Fig. 3:**
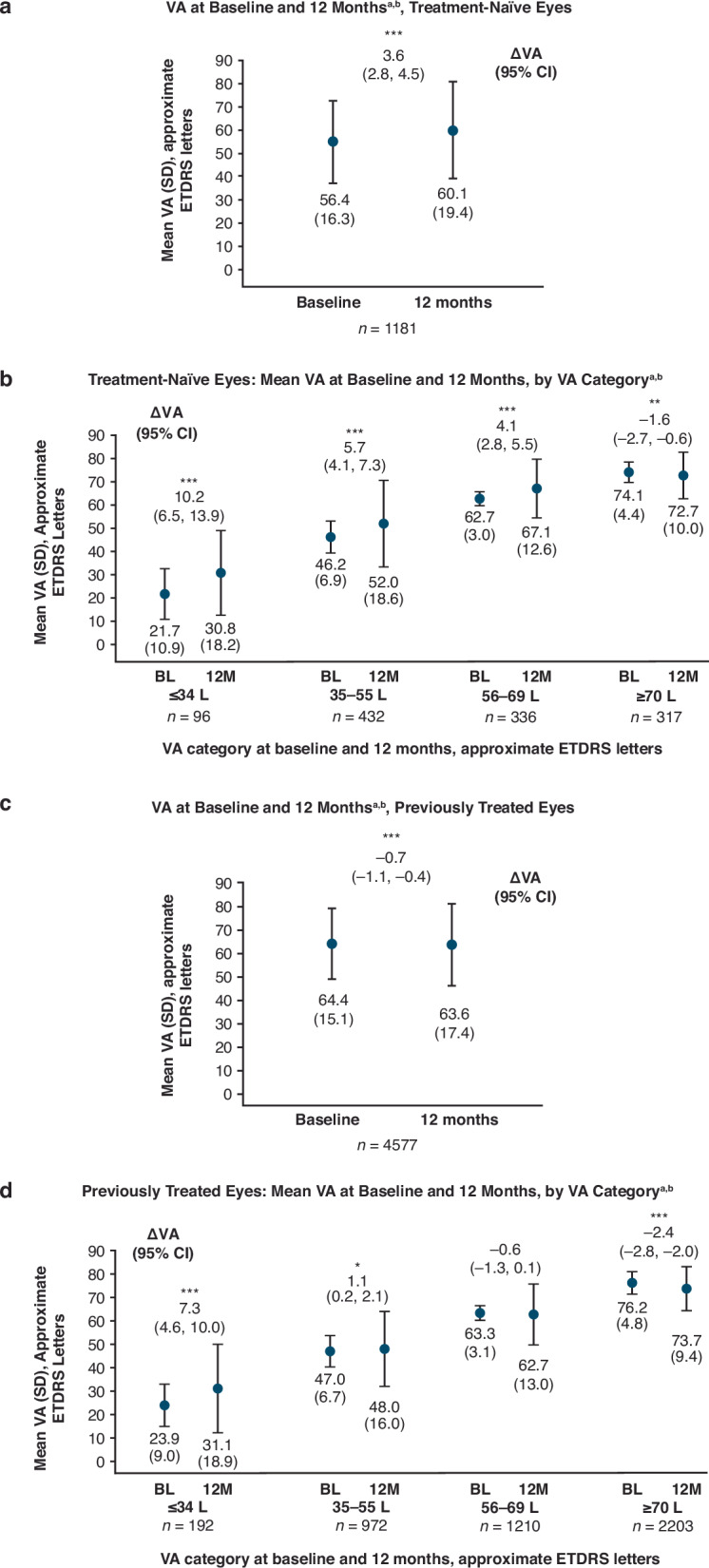
Visual acuity at 12 months since faricimab initiation. **a** Mean VA at baseline and month 12 in treatment-naïve eyes (ETDRS letters). **b** Mean VA at baseline and month 12 and VA change from baseline to month 12 stratified by baseline VA in treatment-naive eyes. **c** Mean VA at baseline and month 12 in previously treated eyes. **d** Mean VA at baseline and month 12 and VA change from baseline to month 12 stratified by baseline VA in previously treated eyes. * Nominal *p* < 0.05 vs baseline. ** Nominal *p* < 0.01 vs baseline. *** Nominal *p* < 0.001 vs baseline. ^a^ Among eyes with VA data at baseline and 12-month timepoint. ^b^ Baseline corresponds with the first faricimab injection. CI confidence interval, ETDRS Early Treatment Diabetic Retinopathy Study, L letter, SD standard deviation, VA visual acuity.

In treatment-naïve eyes, mean (SD) VA increased to 60.1 (19.4) letters at 12 months (mean change from baseline +3.6 letters; 95% CI 2.8, 4.5) (Fig. 3a). Mean changes in VA (95% CI) from baseline at 12 months for eyes with a VA of ≤34, 35–55, 56–69, and ≥70 letters at baseline were +10.2 (+6.5, +13.9), +5.7 (+4.1, +7.3), +4.1 (+2.8, +5.5), and −1.6 (−2.7, −0.6) letters (Fig. 3b). The percentage of eyes with VA ≥70 letters increased to 42% at 12 months.

In previously treated eyes, mean (SD) VA was 63.6 (17.4) letters at 12 months (mean change −0.7 letters; 95% CI −1.1, −0.4) (Fig. 3c). Mean (95% CI) change in VA from baseline at 12 months for eyes with a VA of ≤34, 35–55, 56–69, and ≥70 letters at baseline was +7.3 (+4.6, +10.0), +1.1 (0.16, 2.1), −0.6 (−1.3, 0.1), and −2.4 (−2.8, −2.0) letters, respectively (Fig. 3d). The percentage of eyes with VA ≥70 letters remained stable between baseline and 12 months (48.8%).

Amongst treatment-naïve eyes with VA <70 letters at baseline, 31.3% (*n* = 269/864) attained VA ≥70 letters at 12 months. 20.6% of previously treated eyes with VA <70 letters at baseline (*n* = 488/2374) attained VA ≥70 letters at 12 months. Results stratified by baseline VA are shown in Supplementary Table 2. 71.6% of treatment-naïve eyes (*n* = 227/317) and 79.2% of previously treated eyes (*n* = 1744/2203) maintained VA ≥70 ETDRS letters after 12 months of follow-up.

Amongst treatment-naïve eyes at 12 months, 32.9% (*n* = 389/1181) gained ≥10 ETDRS letters and 20.4% (*n* = 240/1179) gained ≥15 letters; 85.8% (*n* = 1000/1165) avoided loss of ≥10 letters and 90.4% (*n* = 1052/1164) avoided loss of ≥15 letters. Amongst previously treated eyes at 12 months, 12.8% (*n* = 587/4575) gained ≥10 letters and 7.0% (*n* = 318/4563) gained ≥15 letters, and 85.2% (*n* = 3886/4560) avoided loss of ≥10 letters and 91.3% (*n* = 4160/4556) avoided loss of ≥15 letters (Supplementary Table 3).

### Safety

Intraocular inflammation and presumed infectious endophthalmitis were assessed among all 11,139 treatment-naïve and 15,013 previously treated eyes included in FARWIDE-nAMD/DMO studies, with any follow-up duration since faricimab initiation. In treatment-naïve eyes, 81 intraocular inflammation and 16 presumed infectious endophthalmitis events occurred amongst 57,641 injections. Intraocular inflammation event rate per injection was 0.14% (95% CI, 0.11%, 0.17%) and presumed infectious endophthalmitis event rate was 0.03% (95% CI, 0.02%, 0.05%).

In previously treated eyes, 137 intraocular inflammation and 44 presumed infectious endophthalmitis events occurred amongst 100,741 injections. Intraocular inflammation event rate per injection was 0.14% (95% CI, 0.11%, 0.16%) and presumed infectious endophthalmitis event rate was 0.04% (95% CI, 0.03%, 0.06%) (Table 2).

**Table 2:** Intraocular inflammation and presumed infectious endophthalmitis among nAMD and DMO eyes.

	Treatment-naïve eyes	Previously treated eyes
Number of injections^a^	57,641	100,741
Events^a–c^		
Intraocular inflammation, *n*	81	137
% (95% CI)	0.14 (0.11, 0.17)	0.14 (0.11, 0.16)
Presumed infectious endophthalmitis, *n*	16	44
% (95% CI)	0.03 (0.02, 0.05)	0.04 (0.03, 0.06)

*AE* adverse event, *CI* confidence interval, *DMO* diabetic macular oedema, *nAMD* neovascular age-related macular degeneration, *VEGF* vascular endothelial growth factor.

^a^Among all 11,139 treatment-naïve and 15,013 previously treated eyes meeting the eligibility criteria of the FARWIDE-nAMD/DMO studies. AEs are included if they are within 180 days after a faricimab injection and before a subsequent anti-VEGF injection. If AEs of the same type occur within 30 days of each other, then only the first is included. Only one event per injection is counted.

^b^Intraocular inflammation and endophthalmitis (presumed infectious endophthalmitis) events were identified using the post-operative complications, diagnoses or clinical examination findings on the patient record in the electronic medical record. Endophthalmitis events were additionally identified using surgical records of vitreous biopsy, anterior chamber tap or intravitreal treatments of ceftazidime or vancomycin on the patient’s electronic medical record. A detailed list of diagnoses, clinical exam findings and post-operative complications relating to intraocular inflammation or endophthalmitis is provided in Supplementary Table 4.

^c^The exact χ² method was used to calculate the 95% CI for the rates.

## Discussion

FARWIDE is the largest real-world data study outside the US evaluating faricimab treatment patterns and visual outcomes among treatment-naïve and previously treated eyes. FARWIDE-nAMD showed that after 12 months of faricimab treatment, mean VA improved by approximately four letters in treatment-naïve eyes and remained stable in previously treated eyes. One third of eyes gained two lines of vision (32.9%), and 20.4% gained three lines of vision at 12 months. Furthermore, the proportion of treatment-naïve and previously treated eyes with driving level VA (≥70 letters) increased over 12 months, from 26.8% to 42.0% in treatment-naïve eyes and remained stable (48.1% to 48.8%) in previously treated eyes.

The mean number of injections reduced substantially in months 7–12 compared with months 1–6 of treatment, almost halving in treatment-naïve eyes, suggesting treatment interval extension. Intraocular inflammation and presumed infectious endophthalmitis rates were low and aligned with those observed in phase 3 faricimab trials and rates reported in other real-world studies of faricimab [14, 15, 19, 21, 29]. This study shows that faricimab has the potential to extend treatment intervals while improving vision in naïve eyes and preventing vision loss in eyes switched to faricimab from anti-VEGF treatments in the real-world setting.

The phase 3 TENAYA/LUCERNE clinical trials in nAMD demonstrated vision benefits with faricimab in treatment-naïve patients over 1 year [14]. Nominal VA gains were observed in treatment-naïve eyes, suggesting that faricimab can improve vision in the real-world context. Notably, previously treated patients were not included in TENAYA/LUCERNE, so FARWIDE-nAMD provides essential data that are currently lacking in this population.

Several small, single-centre studies have found results comparable to FARWIDE, with vision improvements observed in treatment-naïve eyes and maintenance of vision in previously treated eyes. A retrospective UK study investigated faricimab treatment of 98 eyes from 79 patients who had responded sub-optimally to aflibercept 2.0 mg. In these eyes, vision remained stable during the loading phase [21]. Another case series from the UK, including 81 previously treated eyes from 68 patients, also showed stable VA over time [20]. A consecutive case series of 127 patients with nAMD or DMO (including 74 eyes with nAMD, 35 treatment-naïve and 39 previously treated) in the UK found numerical improvements in VA with faricimab treatment in treatment-naïve eyes after loading [18]. A retrospective study of 107 previously treated eyes with nAMD showed that mean VA remained stable over 6 months [29]. Finally, a small retrospective UK study of three treatment-naïve and eight previously treated eyes noted VA improvements after 1 month of treatment [19].

In FARETINA-nAMD, an extensive registry study from the US including 2184 treatment-naïve eyes and 26,851 previously treated eyes, mean VA increased from 57.3 letters at first faricimab injection to 62.0 letters at the seventh faricimab injection in treatment-naïve eyes and remained stable in previously treated eyes over the same range of injections [30, 31].

The finding from FARWIDE-nAMD that mean injections are reduced in months 7–12 of treatment versus months 1–6 suggests that faricimab treatment intervals are being extended following the initial loading phase. The relatively high number of mean injections in the first 12 months is likely owing to adherence to a standard loading course of faricimab (one injection per month for 4 months) in a high proportion of eyes in FARWIDE-nAMD. In FARETINA-nAMD, mean injection frequency was also higher in months 1–6 vs 7–12 in both treatment-naïve (4.0 vs 2.4) and previously treated (4.2 vs 3.2) eyes. Despite potential differences between the UK and US owing to different protocol adherence, this finding suggests extensions are possible with faricimab in real-world contexts and may be contributing to the lower injection count in months 7–12 of faricimab treatment [31]. Recent single-centre UK studies of previously treated eyes receiving faricimab found that 42.9% patients were on ≥8-weekly intervals after 1 year of follow-up [32], and 28% on intervals ≥10 weeks [21], further supporting real-world faricimab durability.

Durability is a key target for newer nAMD therapies, aiming to reduce the treatment burden on patients and caregivers and potentially increase healthcare system capacity, particularly in resource-constrained healthcare systems such as those in the UK [33, 34]. Indeed, a recent cost–benefit analysis of faricimab in nAMD treatment in Sweden found that although faricimab medication costs are higher than anti-VEGF monotherapies, the reduced frequency of appointments over long-term use reduced visit costs for the healthcare system and travel costs for patients, thereby improving overall cost-effectiveness [35]. A modelling approach based on a resource-constrained UK hospital suggested that over 5 years, faricimab could potentially save £15 million GBP in costs compared with aflibercept 2.0 mg and minimise delayed injections compared with both aflibercept 2.0 mg and ranibizumab biosimilar [34]. Our real-world findings of reduced injection frequency over time suggest that faricimab could provide an option to improve current clinic capacity; however, comparisons to other treatments would be needed to confirm this.

Intraocular inflammation and presumed infectious endophthalmitis rates in the overall FARWIDE population were low, aligning with rates observed in clinical trials [14, 15]. These rates are also aligned with those from published real-world studies [19, 21, 36, 37]. TRUCKEE reported similarly low rates of intraocular inflammation (0.02%) and endophthalmitis (0.04%) in eyes receiving a total of 11,450 injections, with no cases of retinal vasculitis or artery occlusion [30]. Results from FARETINA also report low rates of intraocular inflammation and endophthalmitis per injection in a cohort of DMO and nAMD eyes (intraocular inflammation 0.12%, endophthalmitis 0.07%) [31]. Furthermore, these studies found no evidence of additional safety signals for faricimab in routine clinical practice for intraocular inflammation and presumed infectious endophthalmitis.

FARWIDE-nAMD has several strengths. The heterogeneous patient population with diverse treatment backgrounds is more representative of patients with nAMD encountered in clinical practice than trial patients [14, 15]. The findings will have wide applicability in the UK NHS context because of the multi-centre and multi-region study design. FARWIDE has a high proportion of treatment-naïve eyes, giving an opportunity to understand how faricimab treatment influences patient outcomes in a non-US population and the real-world impact of first-line faricimab use on outcomes in patients with nAMD. Moreover, clinical trials have not evaluated faricimab use in previously treated eyes, so FARWIDE provides an opportunity to assess faricimab outcomes in these patients.

Like other real-world studies, there are limitations to the data presented here. Data are limited to those collected in routine practice. Deceased markers may not be a true reflection of the patient’s vitality status, depending on the Patient Administration System used by the site and its interface with the Medisoft electronic medical records. VA measurements were not standardised; logMAR and Snellen scores were also used and converted into approximate ETDRS letters for analysis. Furthermore, reported data are UK and NHS-specific and may not readily apply to other locations or UK private practice. Finally, no anatomical data were collected, so the impact of faricimab on retinal fluid clearance in this population is unknown. However, the ability to reduce injection frequency following loading suggests better retinal drying as physicians are unlikely to increase treatment intervals for patients who do not have a dry retina, based on faricimab treatment protocols [38]. Multiple studies have shown rapid initial improvements in central subfield thickness (CST) and other anatomical outcomes during the loading phase, including intraretinal and subretinal fluid, pigment epithelial detachment, and diffuse retinal thickening. These include UK single-centre studies [18–21, 29] and larger studies from the US [30, 39]. TRUCKEE showed CST reductions after three faricimab injections in treatment-naïve and previously treated eyes. CST reductions were also observed in patients-eyes in FARETINA-nAMD over seven injections [31].

In conclusion, FARWIDE-nAMD adds to the growing real-world evidence regarding faricimab treatment patterns, effectiveness, and safety. Treatment-naïve eyes gained vision and previously treated eyes maintained vision through 12 months of follow-up. Reduction in mean injections in months 7–12 highlights the potential real-world extended durability of faricimab. Switching to faricimab may reflect a desire among patients and ophthalmologists to seek treatment benefits not fully achieved by previous anti-VEGF monotherapy, including stabilisation of vision or reduced treatment burden. Longer-term effectiveness and safety outcomes will be evaluated in future analyses of this ongoing study.

## Summary

### What is known about this topic

- Faricimab is a novel bispecific antibody targeting both vascular endothelial growth factor-A and angiopoietin 2. It has demonstrated vision gains and improved anatomical outcomes in patients with neovascular age-related macular degeneration (nAMD) in two phase 3 clinical trials. By targeting both cytokines, faricimab has the potential to reduce treatment burden by extending the injection interval compared to standard anti-VEGF therapies.
- Outcomes from treatments in daily practice may not match those observed in clinical trials, due to heterogeneous patient characteristics, disease severity, and adherence level
- It is crucial to investigate the clinical effectiveness and safety of novel treatments in the real-world setting to support physicians in making appropriate treatment choices for their patients.

### What this study adds

- FARWIDE is the largest real-world study of faricimab use outside of the USA, and is investigating the effectiveness, safety and durability of faricimab amongst patients with nAMD and DMO from UK NHS clinics using data from the Medisoft electronic medical record systems.
- In FARWIDE-nAMD, data from patients with 12 months of follow-up after their first faricimab injection shows that treatment-naïve eyes had improved vision from baseline, while eyes that had previously received anti-VEGF treatment had stable vision from baseline to 12 months. The injection frequency sharply declined in the second six months of treatment compared to the first six months, and rates of intraocular inflammation and presumed infectious endophthalmitis were similar to those in phase 3 clinical trials of faricimab.
- FARWIDE-nAMD highlights the real-world effectiveness and safety of faricimab, with the reduction in injection number after the first six months of treatment suggesting that faricimab may be a durable treatment for nAMD in daily practice. This could help alleviate the treatment burden for patients with nAMD.

## Supplementary information

Supplemental Material

## Data Availability

The participating sites did not give written consent for disaggregated data to be shared, so raw data must remain confidential and cannot be made available. Aggregated data are available upon reasonable request.
