# Supplementary material for: Real-world treatment patterns and visual outcomes of faricimab in patients with neovascular age-related macular degeneration in the UK at 12 months: the FARWIDE-nAMD study
Source: Eye (Lond). 2026 Mar 10;40(8):1128–37. doi: 10.1038/s41433-025-04213-2 (PMC13194951; doi:10.1038/s41433-025-04213-2)
Supplement: Supplementary file 1 — Supplemental Material [file 41433_2025_4213_MOESM1_ESM.docx]

# **SUPPLEMENT**

This supplement includes additional details on methods, as well as supplementary tables providing expanded data on select results and methods. This section supports the main text by providing further details into the study’s analyses and findings.

# **SUPPLEMENTARY METHODS**

## **Data source**

The Medisoft electronic medical record systems are used to record ophthalmic assessments, treatments, and diagnostic information at over 200 hospitals in the United Kingdom and are designed to capture highly structured clinical data, which allows for the collation of data from multiple sites to determine real-world clinical outcomes.

## **Index of Multiple Deprivation (IMD)**

The unit areas used to calculate IMD in England are Lower layer Super Output Areas (LSOAs).^1^ In Scotland, the unit areas used were Data Zones.^2^ The deciles are calculated by ranking the 32,844 LSOAs in England and 6976 Data Zones in Scotland. Areas in England and Scotland are ranked separately. IMD were categorised into decile categories 1–2, 3–4, 5–6, 7–8, and 9–10.

## **Additional visual acuity outcomes**

The semi-quantitative values of count fingers, hand motion, perception of light, and no perception of light was converted to 0 Early Treatment Diabetic Retinopathy Study (ETDRS) letter score for the purposes of the analysis^3^ as were any logMAR values worse than 1.68. A correction priority order was implemented to reduce potential bias if a different correction was used at baseline and follow-up intervals for any given patient. At each time point, the best value between habitual correction visual acuity (e.g. glasses, contact lens, refraction test) and unaided visual acuity was used. Where no results are available for either of these measures, pinhole visual acuity was used.^4^

## **Prior anti-VEGF treatments**

Anti-vascular endothelial growth factor (VEGF) agents used before faricimab were categorised as aflibercept 2 mg, brolucizumab, bevacizumab, ranibizumab, and biosimilars.

## **Faricimab treatment characteristics**

Duration of faricimab follow-up was defined as the time between baseline and either the last visit before the data extraction or the date of the first non-faricimab anti-VEGF injection after baseline, where present.

## **Statistical analysis**

Data were summarised descriptively and expressed as mean (standard deviation [SD]) for continuous variables or as the number of eyes/patients (*n*) (%) for categorical variables. Mean changes in visual acuity (ETDRS letters) corresponding 95% confidence intervals were analysed using generalised estimating equations. Nominal *p-*values were calculated as appropriate. As *p-*values are nominal and not adjusted for multiplicity, no formal statistical conclusion should be made based on these. Results were analysed separately for treatment-naïve and previously treated eyes. Data modelling was conducted using the application Structured Query Language (SQL) Server Management Studio (SSMS), and data were analysed using the statistical software R with the integrated development environment RStudio (SQL Server version: SQL Server 2019 Enterprise; SSMS version: SQL Server Management Studio version: 20.1; R version: 4.4.0; RStudio version: 2024.04.0+735).

## **Adverse event analysis**

Intraocular inflammation and presumed infectious endophthalmitis events were identified using the post-operative complications, diagnoses, or clinical examination findings on a patient’s electronic medical record. Any endophthalmitis was excluded from intraocular inflammation. The full list of clinical terms and procedures used to identify intraocular inflammation and presumed infectious endophthalmitis are detailed in Supplementary Table 4.

Rates were evaluated for all eyes with neovascular age-related macular degeneration and diabetic macular edema in FARWIDE with any duration of follow-up since baseline. This was done to ensure that all available safety data on faricimab from the FARWIDE study was reported, rather than safety data from select subpopulations only. Study eyes were observed for adverse events from the date of the first faricimab injection (baseline) until the study eye received an anti-VEGF injection other than faricimab or until the date of data extraction if the former did not occur. All events occurring during this time were evaluated.

**REFERENCES**

1. UK Government. English Indices of Deprivation 2019. The English Indices of Deprivation 2019 (IoD 2019). 2019. <https://assets.publishing.service.gov.uk/media/5d8b399a40f0b609946034a4/IoD2019_Infographic.pdf>.
2. Scottish Government. Scottish Index of Multiple Deprivation (SIMD) 2020. 2020. <https://www.spatialdata.gov.scot/geonetwork/srv/eng/catalog.search#/metadata/02866b0b-66e5-46ab-9b1c-d433dc3c2fae>.
3. Norridge CFE, Gruska-Goh MH, McKibbin M, Donachie PHJ. National Ophthalmology Database Audit 2023. 2023. <https://nodaudit.org.uk/sites/default/files/2023-02/NOD%20AMD%20Audit%20Full%20Annual%20Report%202023_0.pdf>.
4. Jaycock P, Johnston RL, Taylor H, Adams M, Tole DM, Galloway P, et al. The Cataract National Dataset electronic multi-centre audit of 55,567 operations: updating benchmark standards of care in the United Kingdom and internationally. Eye (Lond). 2009;23:38–49.

## **Supplementary Table 1.** Participating NHS trusts.

| **Participating NHS trusts** |
| --- |
| Barking, Havering and Redbridge University Hospitals NHS Trust |
| Bedford Hospital NHS Trust |
| Bradford Teaching Hospitals NHS Foundation Trust |
| Buckinghamshire Healthcare NHS Trust |
| Calderdale and Huddersfield NHS Foundation Trust |
| Colchester Hospital University NHS Foundation Trust |
| East Cheshire NHS Trust |
| East Sussex Healthcare NHS Trust |
| Gloucestershire Hospitals NHS Foundation Trust |
| Great Western Hospitals NHS Foundation Trust |
| Hull and East Yorkshire Hospitals NHS Trust |
| Isle of Wight NHS Trust |
| Leeds Teaching Hospitals NHS Trust |
| Liverpool University Hospitals NHS Foundation Trust |
| London North West Healthcare NHS Trust |
| Mid Cheshire Hospitals NHS Foundation Trust |
| Mid Yorkshire Hospitals NHS Trust |
| NHS Grampian |
| NHS Highland |
| Oxford University Hospitals NHS Foundation Trust |
| Royal Berkshire NHS Foundation Trust |
| Royal Cornwall Hospitals NHS Trust |
| Royal Free London NHS Foundation Trust |
| Salisbury NHS Foundation Trust |
| Sheffield Teaching Hospital NHS Foundation Trust |
| South Tees Hospitals NHS Foundation Trust |
| South Warwickshire NHS Foundation Trust |
| Taunton and Somerset NHS Foundation Trust |
| The Hillingdon Hospital NHS Foundation Trust |
| The Newcastle Upon Tyne Hospitals NHS Foundation Trust |
| Torbay and South Devon NHS Foundation Trust |
| University Hospital Southampton NHS Trust |
| University Hospitals Bristol NHS Foundation Trust |
| Warrington and Halton Hospitals NHS Foundation Trust |
| Wrightington, Wigan and Leigh NHS Foundation Trust |

## **Supplementary Table 2.** Patients gaining and maintaining VA ≥70 ETDRS letters at 12 months in the FARWIDE-nAMD 12-month cohort.

| **Treatment-naïve patient-eyes** | **Eyes in category at baseline, *n*** | **Attaining or maintaining eyes, *n* (%)** |
| --- | --- | --- |
| **Attainment of ≥70 ETDRS letters** |  |  |
| VA <70 ETDRS letters at baseline | 864 | 269 (31.3) |
| VA ≤34 ETDRS letters at baseline | 96 | 3 (3.1) |
| VA 35-55 ETDRS letters at baseline | 432 | 91 (21.1) |
| VA 56-69 ETDRS letters at baseline | 336 | 175 (52.1) |
| **Maintenance of VA ≥70 ETDRS letters** |  |  |
| VA ≥70 ETDRS letters at baseline | 317 | 227 (71.6) |
| **Previously treated patient-eyes** | **Qualifying eyes, *n*** | **Attaining or maintaining eyes, *n* (%)** |
| **Attainment of ≥70 ETDRS letters** |  |  |
| VA <70 ETDRS letters at baseline | 2374 | 488 (20.6) |
| ≤34 ETDRS letters at baseline | 192 | 10 (5.2) |
| 35–55 ETDRS letters at baseline | 972 | 94 (9.7) |
| 56–69 ETDRS letters at baseline | 1210 | 384 (31.7) |
| **Maintenance of ≥70 ETDRS letters** |  |  |
| VA ≥70 ETDRS letters at baseline | 2203 | 1744 (79.2) |

*ETDRS* Early Treatment Diabetic Retinopathy Study, *nAMD* neovascular age-related macular degeneration, *VA* visual acuity.

## **Supplementary Table 3.** Patients gaining or avoiding loss of ≥10 or ≥15 ETDRS letters at 12 months in the FARWIDE-nAMD 12-month cohort.

| **Treatment-naïve patient-eyes** | **Qualifying eyes, *n*** | **Eyes gaining/avoiding loss of VA, *n* (%)** |
| --- | --- | --- |
| Gain of ≥10 ETDRS letters VA | 1181^a^ | 389 (32.9) |
| Avoiding loss of ≥10 ETDRS letters VA | 1165^b^ | 1000 (85.8) |
| Gain of ≥15 ETDRS letters VA | 1179^c^ | 240 (20.4) |
| Avoiding loss of ≥15 ETDRS letters VA | 1164^d^ | 1052 (90.4) |
| **Previously treated patient-eyes** | **Qualifying eyes, *n*** | **Eyes gaining/avoiding loss of VA, *n* (%)** |
| Gain of ≥10 ETDRS letters VA | 4575^a^ | 587 (12.8) |
| Avoiding loss of ≥10 ETDRS letters VA | 4560^b^ | 3886 (85.2) |
| Gain of ≥15 ETDRS letters VA | 4563^c^ | 318 (7.0) |
| Avoiding loss of ≥15 ETDRS letters VA | 4556^d^ | 4160 (91.3) |

^a^ Eyes with ≤90 letters at baseline. ^b^ Eyes with ≥10 ETDRS letters at baseline. ^c^ Eyes with ≤85 letters at baseline. ^d^ Eyes with ≥15 ETDRS letters at baseline. Percentage of eyes avoiding loss of ≥10 or ≥15 ETDRS letters is defined as ([total qualifying eyes – eyes losing vision]/qualifying eyes) × 100. *ETDRS* Early Treatment Diabetic Retinopathy Study, *nAMD* neovascular age-related macular degeneration, *VA* visual acuity.

## **Supplementary Table 4.** Diagnoses, clinical examination findings, post-operative complications and procedures used to identify intraocular inflammation and presumed infectious endophthalmitis following an injection.

| **Diagnoses (intraocular inflammation)** | **Associated ICD-10 code** |
| --- | --- |
| AC cells 0.5+; AC cells 1+; AC cells 2+; AC cells 3+; AC cells 4+; acute anterior uveitis; anterior uveitis; iritis | H20.0 |
| Chronic anterior uveitis | H20.1 |
| Drug-induced uveitis; fibrinous uveitis; idiopathic uveitis; intermediate uveitis; iridocyclitis; post-operative uveitis; uveitis; vitritis | H20.9 |
| Choroiditis involving the macula; retinitis involving the macula | H30.0 |
| Diffuse choroiditis | H30.1 |
| Pars planitis | H30.2 |
| Chorioretinitis; chorioretinitis involving the macula; choroiditis; posterior uveitis; retinitis | H30.9 |
| Perivascular infiltrate; retinal vasculitis; retinal vasculitis – mixed arteritis and periphlebitis; retinal vasculitis – non-occlusive; retinal vasculitis – occlusive; retinal vasculitis – periphlebitis / venous sheathing; retinal vasculitis – predominantly arteritis; vascular sheathing | H35.0 |
| 1+ vitreous inflammation; 2+ vitreous inflammation; 3+ vitreous inflammation; 4+ vitreous inflammation; trace vitreous inflammation; vitreous cells present; vitreous haze 0.5+; vitreous haze 1+; vitreous haze 2+; vitreous haze 3+; vitreous haze 4+; vitreous inflammation; vitreous inflammation – no red reflex; vitreous inflammation – red reflex present | H43.8 |
| Panuveitis | H44.1 |
| **Post-operative complications (intraocular inflammation)** | **Associated ICD-10 code** |
| anterior uveitis; post-operative uveitis; retinal vasculitis; vitritis | N/A |
| **Diagnoses (presumed infectious endophthalmitis)** | **Associated ICD-10 code** |
| 1 mm hypopyon; 2 mm hypopyon; 3 mm hypopyon; 4 mm hypopyon; AC cells 0.5+; AC cells 1+; AC cells 2+; AC cells 3+; AC cells 4+; hypopyon; trace hypopyon | H20.0 |
| Secondary open angle glaucoma (acute anterior uveitis) | H40.4 + H20.0 |
| Secondary open angle glaucoma (panuveitis) | H40.4 + H44.1 |
| Post-operative endophthalmitis | H44.0 |
| Endophthalmitis; exogenous fungal endophthalmitis; panuveitis | H44.1 |
| **Post-operative complications (presumed infectious endophthalmitis)** | **Associated ICD-10 code** |
| Endophthalmitis; hypopyon | N/A |
| **Procedures (presumed infectious endophthalmitis)** | **Associated OPCS-4 code** |
| Anterior chamber tap | C69.8 |
| Intravitreal injection (specified agents: amikacin 0.4 mg + vancomycin 1.0 mg in 0.2 ml; ceftazidime 2.2 mg/0.1 ml; ceftazidime 2.25 mg/0.1 ml; vancomycin 1 mg/0.1 ml; vancomycin 2 mg/0.1 ml; vancomycin 5 mg/0.5 ml) | C79.4 |
| Vitreous biopsy | C79.8 |

There are some terms in the Medisoft library (AC cells, panuveitis) which, if recorded on their own, could infer that either an intraocular inflammation or a presumed infectious endophthalmitis event occurred. These terms were assumed to indicate that an intraocular inflammation event occurred unless recorded alongside another presumed infectious endophthalmitis term.

*AC* anterior chamber; *ICD-10* International Classification of Diseases 10th Revision, N/A, not applicable.
